# Acoustic Trapping and Manipulation of Hollow Microparticles under Fluid Flow Using a Single-Lens Focused Ultrasound Transducer

**DOI:** 10.1021/acsami.3c11656

**Published:** 2023-11-02

**Authors:** Paul Wrede, Amirreza Aghakhani, Ugur Bozuyuk, Erdost Yildiz, Metin Sitti

**Affiliations:** † Physical Intelligence Department, Max Planck Institute for Intelligent Systems, 70569 Stuttgart, Germany; ‡ Institute of Biomaterials and Biomolecular Systems, University of Stuttgart, 70569 Stuttgart, Germany; § Institute for Biomedical Engineering, ETH Zurich, 8092 Zurich, Switzerland; ∥ School of Medicine and School of Engineering, Koç University, Istanbul, 34450, Turkey

**Keywords:** acoustic manipulation, hollow microparticles, acoustic trapping, focused ultrasound, microrobotics, particle manipulation, ultrasound imaging, microbubbles

## Abstract

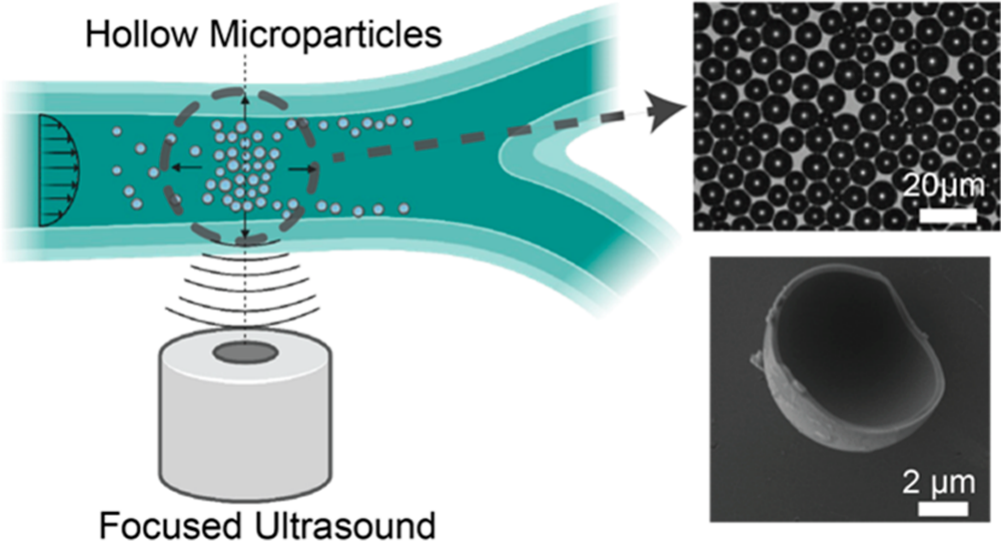

Microparticle manipulation and trapping play pivotal roles in biotechnology. To achieve effective manipulation within fluidic flow conditions and confined spaces, it is necessary to consider the physical properties of microparticles and the types of trapping forces applied. While acoustic waves have shown potential for manipulating microparticles, the existing setups involve complex actuation mechanisms and unstable microbubbles. Consequently, the need persists for an easily deployable acoustic actuation setup with stable microparticles. Here, we propose the use of hollow borosilicate microparticles possessing a rigid thin shell, which can be efficiently trapped and manipulated using a single-lens focused ultrasound (FUS) transducer under physiologically relevant flow conditions. These hollow microparticles offer stability and advantageous acoustic properties. They can be scaled up and mass-produced, making them suitable for systemic delivery. Our research demonstrates the successful trapping dynamics of FUS within circular tubings of varying diameters, validating the effectiveness of the method under realistic flow rates and ultrasound amplitudes. We also showcase the ability to remove hollow microparticles by steering the FUS transducer against the flow. Furthermore, we present potential biomedical applications, such as active cell tagging and navigation in bifurcated channels as well as ultrasound imaging in mouse cadaver liver tissue.

## Introduction

Microparticle manipulation has an important role in advancing various facets of biomedical research including sensing, targeted drug delivery, cell analysis, microsurgery, imaging, and diagnostics.^1−4^ However, effective particle manipulation and trapping in confined spaces and under fluidic flow conditions present a formidable challenge. For instance, in the circulatory systems, high-velocity blood flow rates limit the margination and effective delivery of microparticle-based drug carriers to the target disease area.^5^ While tuning the size and modulus of passive microparticles can increase their margination efficiency,^6^ the active control of their local concentration within specific confined regions remains elusive. To address these issues, researchers have employed active particle manipulation and trapping systems using optical,^3,7^ magnetic,^8,9^ and acoustic forces,^10,11^ as well as microbubbles^12,13^ and microrobots^14,15^ as the stimuli-responsive agents.

Acoustic waves have emerged as a compelling external energy source in biomedical applications due to their ability to penetrate deep tissues and their biocompatible nature. This has led to the development of acoustic tweezers and manipulation tools to displace matter across a versatile size range for many biomedical applications.^16,17^ Microbubbles are one of the widely used ultrasound agents that are used in the biomedical field both for imaging and therapeutic carriers for gene and drug delivery.^12^ As drug carriers, microbubbles can locally deliver the drug molecules with efficient spatial targeting under the application of focused ultrasound (FUS) waves.^16^ For example, researchers have recently shown the two-step process of local aggregation and uncaging of drug-loaded microbubbles to the blood–brain barrier,^18^ reducing the side effects associated with systemic drug administration. However, the attraction between microbubbles limited the duration of the aggregation step, thereby constraining the quantity of the delivered drug molecules. Overall, microbubbles have shown promising results as ultrasound agents for imaging and therapeutics, but they have certain limitations in clinical applications. First, their circulation half-life is very short (less than 5 min),^19^ lessening their ability to deliver prolonged drugs. Next, microbubbles are rapidly broken upon ultrasound exposure, which again limits the extended imaging time and therapeutic tasks.^13^ Furthermore, acoustically driven microbubbles are prone to become unstable due to issues of dissolution (shrinking of bubble size), fragmentation (breaking up into smaller bubbles), and coalescence (merging of smaller bubbles when they are close together). A recent study showed the successful trapping of microbubbles under fluid flow using an advanced acoustic vortex tweezer setup;^20^ however, the microbubble cluster size did not increase after a point and the coalescence of microbubbles remained an obstacle to their aggregation.

The microrobotics field has also shown promising methods for localized medical interventions.^15,21^ The medical microrobots can be navigated inside confined, hard-to-access regions to reach the targeted disease using wireless actuation methods such as magnetic fields,^22^ acoustic waves,^23^ light,^24^ and chemical cues.^25^ Using surface chemistry methods, the microrobot bodies are coated or decorated with a therapeutic payload (e.g., gene or drug molecules). Upon reaching the target tissue, they can release the payload either passively through diffusion or actively by external stimuli (e.g., ultrasound or laser).^14,26^ However, many challenges remain before their practical medical implementation which include, but are not limited to, the cost of mass production, scalability, the ability to withstand high-speed blood flow of the circulatory system, and a strategy for removing the microrobots once their medical tasks are finished.^27^ To overcome the limitations associated with the use of microbubbles and microrobots in stable manipulation and spatial trapping under fluidic flow conditions, it becomes necessary to explore alternative platforms that integrate the advantages offered by both methodologies.

**Figure 1:**
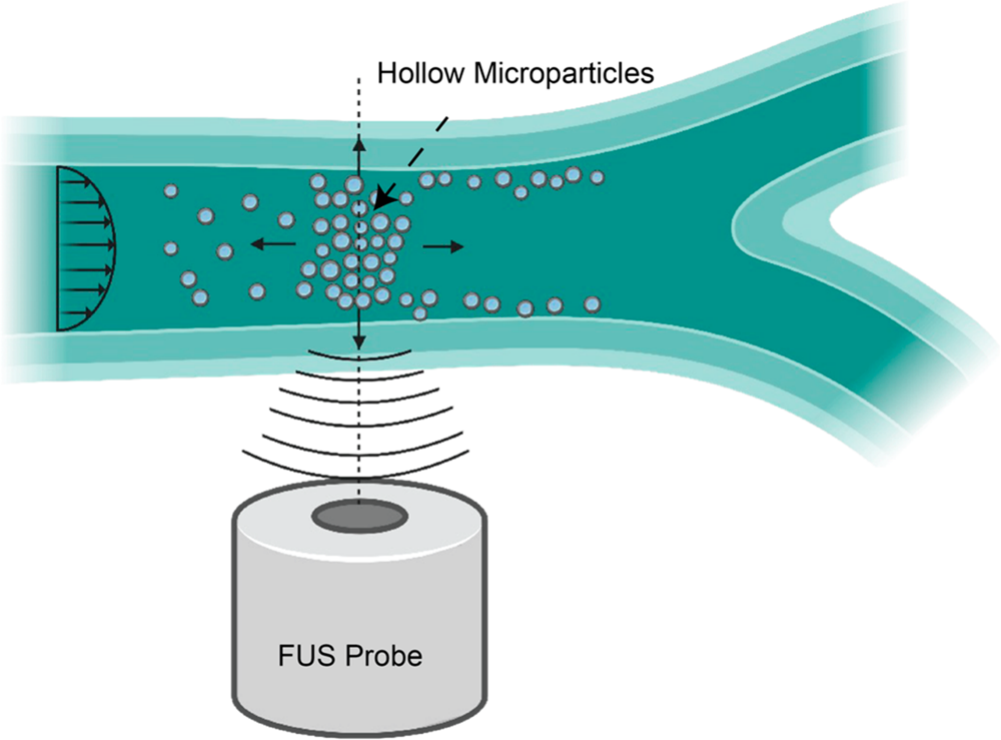
Schematic description of a single-lens FUS transducer trapping hollow particles. The particles (blue) are trapped inside a bifurcated tubing against fluid flow. The single-lens FUS waves used to trap the particles is placed below the tubing. The transmitted focused traveling ultrasound waves apply acoustic trapping force to create a stable aggregate near the focal spot.

In this study, we propose the use of hollow microparticles that can be trapped and manipulated under fluid flow using a single-focus ultrasound wave (Figure 1). These hollow microparticles exhibit a unique combination of properties, merging the benefits of air bubbles found in ultrasound microbubble agents with the structural integrity of synthetic microrobot shell materials. Consequently, they offer exceptional stability and buoyancy, making them well-suited for effective manipulation and trapping in confined environments. Furthermore, their scalability and the ability for mass production enable the injection of a large quantity (>10^6^ microparticles/mL) into the circulatory system. To accomplish acoustic trapping of these hollow microparticles, we employ a single-lens FUS transducer, which eliminates the need for complex acoustic actuation systems such as acoustic vortex tweezers^20,28^ or multiple ultrasonic actuators.^4^ By comparing their acoustic responses to similarly sized solid-filled microparticles, we demonstrate the distinctive acoustic trapping behavior of the hollow microparticles. At the focal spot of FUS waves, the hollow microparticles are robustly trapped inside a vessel under extreme fluid flows, forming stable aggregates through the influence of an acoustic trapping force. Notably, the hollow microparticles maintain their separate bodies, mitigating the coalescence issue encountered with microbubble agents. In addition to trapping capabilities, we showcase the manipulation of the microbubble aggregate inside a vessel and a bifurcated microchannel through the steering of the FUS transducer. Moreover, we present a retrieval procedure by removing most of the microparticles against the fluid flow after injection. Finally, we demonstrate their ultrasound imaging contrast ex vivo and show an active cell tagging process by steering the cluster of hollow microparticles toward tumor spheroids.

## Results

### Physical Characteristics of Hollow Microparticles under Ultrasound Waves

**Figure 2:**
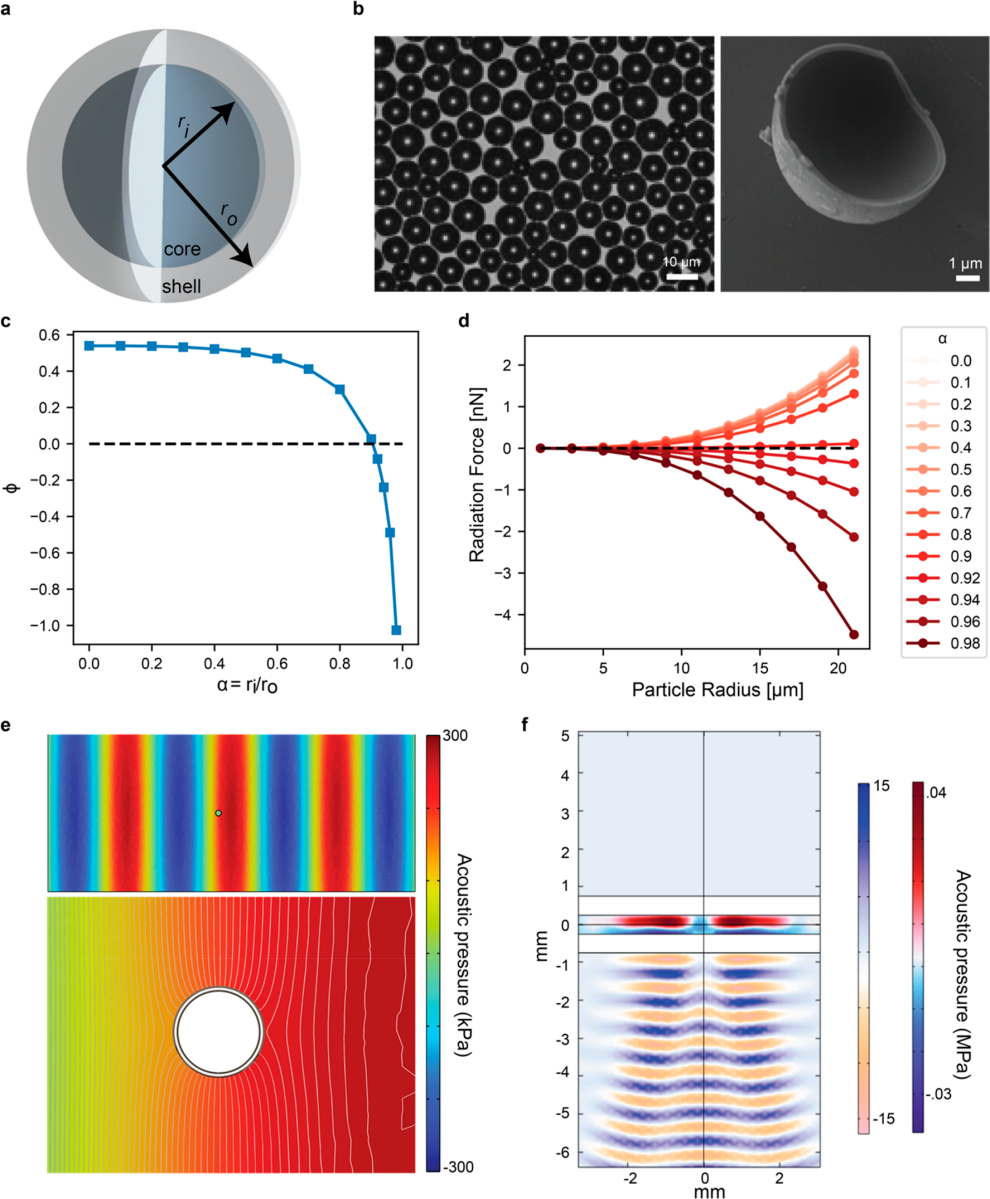
Characterization of the hollow particles and acoustic setup. a) Schematic representation of a hollow core–shell particle with inner radius **r*_i_* and outer radius *r*_*o*_. b) Light microscope image (left) and scanning electron microscope (SEM) image (right) of polydisperse hollow particles. c) This graph shows the acoustophoretic contrast factor for different ratios α of *r*_*i*_ and *r*_*o*_. For small α, as in case of a solid particles, ϕ is positive. When increasing the ratio α the acoustophoretic contrast factor gets highly negative inverting the acoustophoretic behavior of the hollow particles as used for this study compared to solid particles. d) Representation of the acoustic radiation force dependency on the radius of a particle and the ratio α. The graph shows that the radiation force acting on solid particles is positive while it is negative for hollow particles. All values for graphs g) and h) are obtained by numerical simulations.^32^ e) COMSOL finite element simulation of the pressure distribution around a hollow core–shell particle immersed into a standing acoustic wave. f) Finite element simulated pressure applied by a 2 MHz FUS transducer into a 500 μm diameter tubing made from Tygon.

The hollow microparticles consist of a gas-filled core surrounded by a solid borosilicate shell, as shown in Figure 2a. Upon immersion of the dry microparticles inside a fluidic medium, the gas-filled core becomes visible under the microscope as a dark-shaded area due to the light contrast at the water–solid–air interface (Figure 2b). The shell thickness was determined to be around 500 nm, as shown in the scanning electron microscopy (SEM) analysis in Figure 2b. These particles are polydisperse with a size range between 2.5 to 17.5 μm. The sample measurement of a few hundred particles revealed that most particles have a larger size distribution between 8 and 12 μm (Figure S1). The borosilicate particles were chosen due to their commercial availability, allowing for a steady supply of particles with minimal batch-to-batch variations. Additionally, borosilicate is nondissolvable in water and biological fluids such as blood. It also allows for easy functionalization due to the presence of NH groups, as will be demonstrated later. The biocompatibility of borosilicate is unclear. While it is considered safe as an ingredient for cosmetic products,^29^ another study shows its application as an antibacterial material for pharmaceutical packaging when doped with Zn.^30^ We, therefore, conducted initial cell viability studies with immune cells (THP1). Here, borosilicate does not show a toxic effect on cells (Figure S2).

To understand the acoustic properties of the hollow microparticles, the acoustophoretic contrast factor ϕ was calculated assuming a one-dimensional (1D) standing wave and a nonviscous fluidic medium:^31,32^
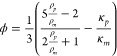
1where *ρ*_*p*_, *ρ*_*m*_, *κ*_*p*_ and *κ*_*m*_ are the density of the particle and medium as well as the compressibility of both the particle and medium, respectively. According to eq 1, the acoustophoretic contrast factor is mainly dependent on the material and geometrical properties of the hollow particle. Additionally, for core–shell-particles, the mean density of the particle *ρ*_*p*_ depends on the particle’s inner and outer radius *r*_*i*_ and *r*_*o*_ as , where . For solid particles, this ratio is 0, while for core–shell particles, it is between 1 and 0. The present hollow borosilicate particles have an α value of about 0.98. As presented in Figure 2c, ϕ decreases with increasing α. Starting from α = 0.9, the acoustophoretic contrast factor becomes 0. When further increasing α, it becomes negative rapidly. Consequently, the particles proposed here have a highly negative acoustophoretic contrast factor. If ϕ > 0, the particles are attracted to the pressure nodes of a standing wave; otherwise, for ϕ < 0, they are attracted to the pressure antinodes. Note that the contrast factor does not depend on the radius value alone, but rather on the ratio of the inner to the outer radius.

Obtaining the contrast factor for the hollow particles, we can find the acoustic radiation force acting on the particle, assuming the Gor’kov potential as^33^
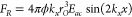
2where *k*_*x*_, *E*_*ac*_, and *x* are the wavenumber, the acoustic energy density, and the spatial position of the particle, respectively. For eq 2, a negative ϕ leads to a negative *F*_*A*_ acting on the particle as shown in Figure 2d. This changes the behavior of the particles inside the acoustic field. While solid spherical particles, which possess positive ϕ, are trapped inside the pressure nodes, the hollow particles with negative ϕ are trapped inside the pressure maxima. Figure 2e depicts the acoustic pressure acting on a single hollow microparticle under a 2 MHz planar ultrasound field. Considering the simulations shown in Figure 2f, the hollow particles are trapped directly in the maximum pressure regions of the FUS focal area. This would enable the precise control of these particles in all axes by using only a single-lens FUS transducer.

Besides the geometric parameters of the hollow particles, the characteristics of the surrounding acoustic field play an important role in their behavior. The acoustic field applied inside a Tygon tubing was measured using a hydrophone, which was inserted into the tubing to measure the applied pressure in three dimensions (3D) around the acoustic focus. The acoustic pressure was applied by a 500 kHz FUS transducer having a focal width of 3.02 mm and focal length of 21.42 mm with a focal depth of 51.74 mm from the exit plane of the probe and a 2 MHz FUS transducer having a focal width of 1.28 mm and a focal length of 15.5 mm with a focal depth of 51.4 mm from the exit plane. The transducers with 63.2 mm radius of curvature for the 500 kHz probe and 55 mm radius of curvature for the 2 MHz probe were positioned on the bottom of the tank. The tubing was positioned inside the transducer focus by using a manual x-y-z stage. A needle hydrophone was used to measure the acoustic pressure amplitude at the focal spot of the FUS waves. For a 150–200 Vpp input voltage, maximum pressure amplitudes of 150–200 kPa were recorded at the focal spot. The simulation results prove that the focus region is characterized by a high positive pressure region surrounded by high negative pressure regions. These two regions were separated by pressure nodes. The following experiments were designed to investigate the predicted trapping behavior of hollow borosilicate particles.

### Acoustic Trapping Performance of Hollow Microparticles under Fluid Flow

**Figure 3:**
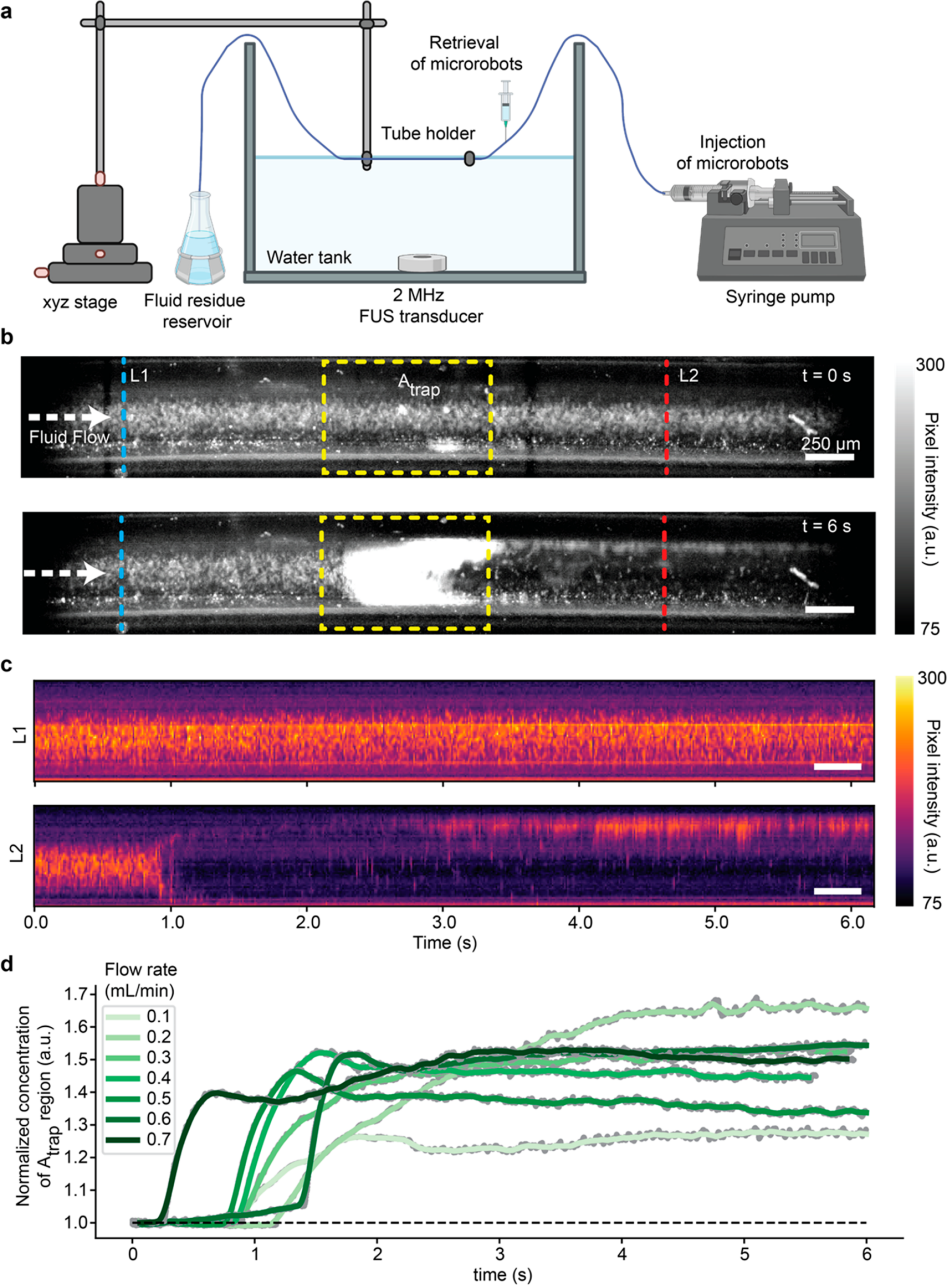
Trapping dynamics of the hollow particles inside a FUS field. a) Schematic depiction of the experimental setup used to evaluate the trapping efficacy of acoustic traveling wave-induced agglomeration of hollow particles inside a 500 μm diameter Tygon tubing. A FUS transducer with a central frequency of 2 MHz is placed inside a water tank. A tubing with a syringe pump was positioned under water using a manual *x*-*y*-*z* stage. The hollow particles were flushed into the tubing using the syringe pump. The liquid was stored in a reservoir. b) Microscope image showing the particles flowing through a Tygon tubing before (top) and after (bottom) applying the FUS. Using a customized Python code different region are defined for automated particle concentrations measurements. The blue dashed line named L1 indicates the cross section of the tubing before the FUS focal spot. The yellow dashed line *A*_*trap*_ and the blue dashed line L2 indicate the trapping region and after trapping region in the flow direction, respectively. c) Time history images of the pixel intensity of the L1 (top) and L2 (bottom) cross section before and after applying the FUS. Both b) and c) refer to an applied flow speed of 25.4 mm/s and a FUS input voltage of 200 mV (peak-to-peak). d) This plot shows the normalized particle concentration inside the trapping region *A*_*trap*_ over time for flow rates reaching from 0.1 to 0.7 mL/min corresponding to the flow speeds of 8.5–59.4 mm/s.

Figure 3a depicts the experimental setup used for testing the acoustic trapping of hollow microparticles at the focal point of the FUS waves. The microparticles were injected into 500 μm-diameter Tygon tubing, at different concentrations of 2.5, 5, and 10 mg/mL, using a syringe pump. We used a 2 MHz FUS transducer and positioned its focal point inside of the tubing. Then, we applied different flow rates and acoustic pressure amplitudes to characterize the trapping performance of microparticles. To determine the trapping behavior, all of the tests were recorded by a high-speed camera (M310; Phantom, Inc.) at 200 or 400 frames per second (fps). The videos were subsequently analyzed by using a custom Python code. Figure 3b shows two representative images for a fluid flow speed of 25.47 mm/s and an input FUS voltage of 200 mV (peak-to-peak) before and after the FUS application (Movie S1). We have defined three regions of interest (ROI) to characterize the trapping efficiency: (1) the blue dashed line (denoted as L1) indicating a cross-section before the focal spot of FUS waves; (2) the yellow dashed square (denoted as *A*_*trap*_) indicating the actual trapping region; and (3) the red dashed line (denoted as L2) representing a cross-section after the trapping region in the direction of the fluid flow. To achieve efficient acoustic trapping, the concentration of particles detected inside the trapping area should increase drastically after ultrasound exposure. The increased microparticle concentration can be clearly seen in the yellow ROI in Figure 3b, where an aggregate of hollow microparticles has formed after 6 s, indicated by a bright white color. Figure 3c shows the time histories of the pixel intensities of the L1 and L2 cross sections. Upon activation of ultrasound at around 0.9 s, the particles after the trapping region (L2) suddenly disappear from the fluid flow and move to the vessel wall, while the continuous stream of particles keeps running with the fluid flow as shown in the L1 time-history map. Note that, after many particles have been trapped at *A*_*trap*_ and a large aggregate has been formed, some particles leaked from the trapping area and moved to the vessel wall boundary after around 3 s. The wall-marginated particles after a few seconds are indicated by the bright pixels at the wall boundary in Figure 3c, L2 time-history map. The rest of the time-history plots for a different range of flow rates and ultrasound amplitudes are shown in Figures S3–S9. To characterize the dynamics of acoustic trapping, we have calculated the sum pixel intensity of *A*_*trap*_ ROI, for different flow speeds from 8.5 to 59.4 mm/s. Figure 3d shows the concentrations of the trapped particles, which are normalized to those before ultrasound application. Immediately after the ultrasound exposure, the concentration sharply increased and reached a steady state, where no more particles were trapped and the local concentration stayed constant. Also, the faster the fluid flow rate, the faster rise of concentration occurred, which could be attributed to the faster rate of incoming particles that were trapped at the focal spot of the FUS wave. Since these observations were made merely based on two-dimensional (2D) images, the calculations would underestimate the actual trapping performance that happened in three-dimensional space. However, the 2D intensity estimations can illustrate the trend of acoustic trapping behavior of hollow microparticles under different fluid flows.

According to our experiments, the trapping efficacy was highly dependent on the input voltage to the transducer, the concentration of particles, and the flow rate of the fluid. Since higher transducer input voltage led to increased pressure in the focal area and consequently a higher acoustic energy density *E*_*ac*_. Consequently, the acoustic trapping force on the particles increased, leading to a better trapping performance. In contrast, an increased flow rate applied a larger drag force on the particles. The closer the drag force magnitude to the acoustic trapping force magnitude, the less effective the acoustic trapping became. Eventually, increasing the drag force could exceed the trapping force, and hence, the acoustic trapping would fail. Besides, the initial concentration of particles was crucial for their effective entrapment at the focal spot of the ultrasound. For our experiments, a larger concentration of particles led to better trapping performance implying that particle–particle interactions, due to the secondary Bjerknes force^34^ and electrostatic interactions, could play an important role. Another parameter presumably strongly influencing the trapping efficiency was the acoustic streaming induced drag force on the particles.^35^ When hollow particles were statically trapped, a clear streaming of particles was detected (Movie S2).

It is worth noting that using FUS for trapping a cluster of microparticles under physiological flow conditions is advantageous compared to other medically prevalent actuation methods, such as magnetic ones. Currently, most microrobot systems rely on magnetic actuation and control.^22,36,37^ It is often favored over other control mechanisms, such as optical, electrical, or chemical, due to its biocompatibility and deep penetration depth.^14,38^ Still, magnetic systems show limitations in terms of heating issues for electromagnets, bulkiness, and poor scalability on the micron scale. Especially the latter presents a major challenge in applications where high forces, such as by fluids, materials, or tissues, need to be overcome.^39^ Using a single FUS transducer to trap particles has advantages over those of state-of-the-art magnetic actuation devices. The FUS system allows for easier access to the body through portable probes and does not require continuous cooling, as electromagnetic systems do. Additionally, the use of ultrasound-responsive hollow microparticles eliminates the need for magnetic coating, reducing biocompatibility concerns near tissues and cells. However, accurately positioning the focal spot inside the body can become complex in areas where reflection and scattering occur near tissues and bones.

In this regard, we performed a basic analysis of the trapping capabilities of acoustic versus magnetic fields. In the context of particle manipulation, magnetic fields are used to trap, form and manipulate nanoparticle swarms for medical applications.^40^ For a thorough overview of the available platforms, we refer to a recent review.^41^ Rotating magnetic fields are commonly used to actuate different types of magnetic microrobots, including the ones locomoting in physiologically relevant flows as used here.^22^ On the other hand, it would not be possible to actuate magnetic particles in the tubing center with such fields.^22^ For example, magnetic particles that are actuated with rotating fields need a nearby wall to perform locomotion. Consequently, the magnetic particles flowing in the vessel center can only be actuated with magnetic field gradients in the scenario given here.

Using simulations, We estimated the drag forces on particles using computational fluid dynamics analyses inside a 500 μm and 4.77 mm diameter pipe as used during experiments. We assumed the trapping region as a single solid particle of 500 μm diameter. This is similar to the size of the trapped particle clusters during our experiments. Additionally, we performed the analysis for particles with a size of 100 and 10 μm to outline the scaling. Depending on the radius of the simulated particle, we moved the particle from the center of the tubing (*z* = 0) toward the pipe wall. The acquired drag forces served as input for a calculation of the magnetic gradient force acting on state-of-the-art hard magnetic L1_0_-FePt thin film-coated particles.^36^Figure S10 shows the required magnetic gradient force to balance the drag force applied to the sphere at the flow speeds used during our experiments. The calculated values were compared to the state-of-the-art magnetic gradients in the literature, a Halbach permanent magnet array,^42^ and the gradients generated in the bore entrance region of a 7 T MRI scanner.^43^ The analysis showed that orders of magnitude higher gradient fields would be needed to balance the drag force acting on the particles. On the contrary, we were able to experimentally manipulate the particles with acoustic trapping in such fluid flows. Even though these simulation results are based on assumptions that might differ from real-world scenarios, acoustic trapping could serve as a promising manipulation technique for biomedical microrobotics applications.

### Acoustic Imaging, Actuation, Manipulation, and Retrieval of Hollow Microparticles

**Figure 4:**
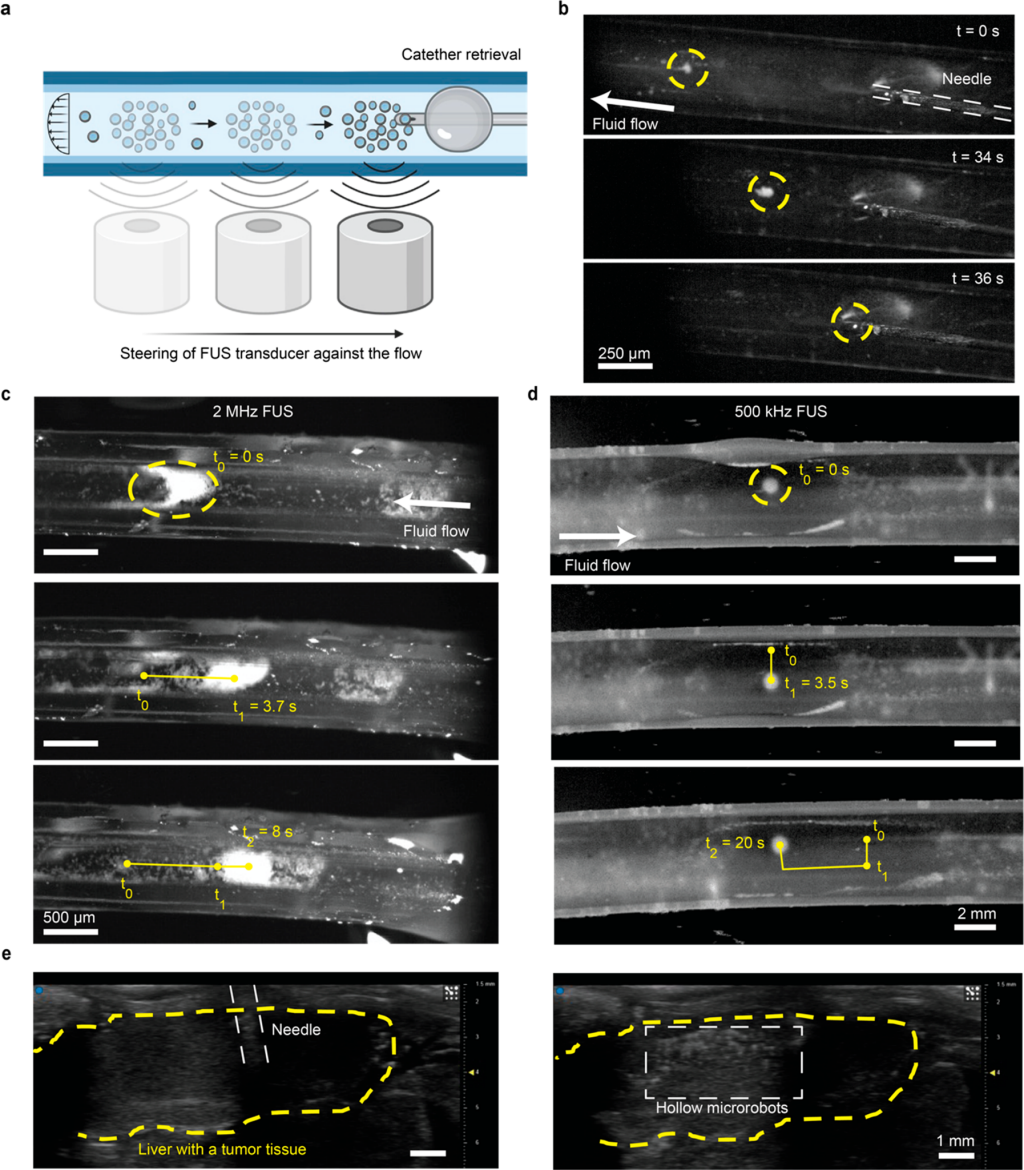
Control and retrieval of the hollow particles against the fluid flow. a) Schematic representation of hollow particles retrieved using acoustic manipulation enabled by a FUS transducer against the fluid flow. b) Time lapse images showing the retrieval of the hollow microparticle swarm immersed into a 4.77 mm-diameter Tygon tubing against a flow speed of 0.93 mm/s. The particles are trapped by using a 500 kHz focused ultrasound transducer. The particles are moved back to the injection side by using a relative movement between the tubing and the transducers. There they are retrieved using the same 28 G needle used for injection. c) Movement of acoustically trapped hollow microparticles inside a 500 μm diameter tubing. Here 100 μL of particles with a concentration of 5 mg/mL are injected into a tubing using a 28 G needle. After injection, the particles are trapped using a 2 MHz focused ultrasound transducer and moved inside the tubing. The particles are imaged using a high-speed camera mounted to a light microscope. The applied flow speed is 25.46 mm/s. d) The particles are trapped using a focused 500 kHz transducer. Using a manual *x-y-z* stage the particles were moved inside the tubing in 2D. The applied flow speed was 4.67 mm/s. For all figures, the trajectories of the particles are marked in blue, and the flow direction is marked in orange. e) Ultrasonographic images of needle positioning (left) and subsequent particle injection (right) into the portal vein of the liver of an ex vivo mouse with multiple liver metastasis. The liver area is marked in yellow. Ultrasound imaging is performed in the B-mode with a frequency of 40 MHz.

Applications at small scales, such as microfluidics or microbiology, require the precise control and actuation of the particles. The same is true for delicate scenarios found in medical applications, where microparticles or drug carriers that are not actively trapped on the target disease region can cause severe side effects such as occlusions. Moreover, retrieval of the microparticles would be beneficial to analyze matter attached to the particles, such as for antibody detection^44^ or to validate vaccine effectiveness.^45^ Also, for microparticle drug delivery systems, such retrieval strategies (e.g., assisted by a catheter) can be beneficial, as illustrated in Figure 4a. One of the major concerns of medical microparticles is their toxicity. Such toxicity depends on many factors, such as the used materials, the location of the application, and the duration.^14,15^ Hence, during the registration process of the U.S. Food and Drug Administration (FDA)^46^ and European Medicines Agency (EMA) for medical devices, higher standards are demanded for all biomedical devices staying inside the body for mid-length or long durations.^47^ Consequently, to improve the biocompatibility of particle-based drug carrier systems and to shorten their future registration processes for use as medical devices, reliable retrieval strategies are necessary.

Motivated by such a requirement, we tested the potential of our acoustic trapping system to retrieve the injected particles from the flow. Here, a 500 kHz focused transducer with a wider focal area was used to demonstrate that trapping was possible with a variety of transducers. For given requirements, the proper probe could be used consequently. During the experiments, the particles were injected into 4.77 mm-diameter Tygon tubing using a 28 G needle. Afterward, the particles were trapped acoustically and moved back to the injection side as shown in Figure 4b. There, the particles were retrieved with the same needle used for injection (Movie S3).

Next, we tested the actuation and control of the particles in confined spaces. For that, a 500 μm-diameter tubing made of Tygon was mounted to a manual *x*-*y*-*z* stage (Thorlabs, USA). This allowed for a relative movement between the tubing and the transducer focus. The applied ultrasound frequency was 2 MHz. The particles immersed in deionized (DI) water were pumped into the tubing by using a syringe pump, which also enabled flow control. The microparticles could be moved inside the tubing filled with along the *y*-axis as shown in Figure 4c. Movie S4 shows the trapping and manipulation of hollow microparticles in DI water and full pig blood mediums. A movement in the *x*-direction was not possible, as the trapped agglomeration of particles fully filled the cross-section of the tubing. The trapping was tested for flow speeds between 8.5 and 59.4 mm/s. The particles could be moved upstream at flow speeds of up to 42.4 mm/s. These flows are similar to those occurring in human veins, enabling the potential use of this technology for vascular applications.^48−50^

To demonstrate control in two dimensions (2D), 4.7 mm diameter tubing was mounted to the *x*-*y*-*z* stage. Using the *x*-*y*-*z* stage, trapped hollow borosilicate particles could be successfully moved around the *x*-*y* plane (Movie S5). The particles could be moved upstream at flow speeds of up to 4.6 mm/s (Figure 4d). The trapping force was not high enough for higher flow speeds to overcome the drag force acting on the particles. Here, a transducer capable of applying a higher pressure could improve the results. The transducer used in these experiments applied pressure of approximately 500 to 600 kPa in the focal region. Also for microfluidic applications in biology, pharmacology, medicine, or chemistry tens of micrometers up to a few centimeters per second flows are expected^51^ which verifies the potential use of this approach in such applications.

Lastly, the hollow particles were injected into an *ex vivo* mouse liver with tumor tissue to demonstrate their biomedical imaging capability in real clinical scenarios. For this purpose, we injected hollow microparticles into the portal vein of a dead mouse with liver metastases. Thanks to their hyperechoic appearance under ultrasound imaging, we observed the distribution of the hollow microparticles in the liver with multiple tumors (Figure 4e, Movie S7). In addition to their acoustic actuation capabilities, their contrast-enhancing behavior in ultrasonography makes them promising candidates for various clinical drug delivery applications (e.g., in oncology).^52^

### Active Cell Targeting and Acoustic Manipulation in Bifurcated Channels

**Figure 5:**
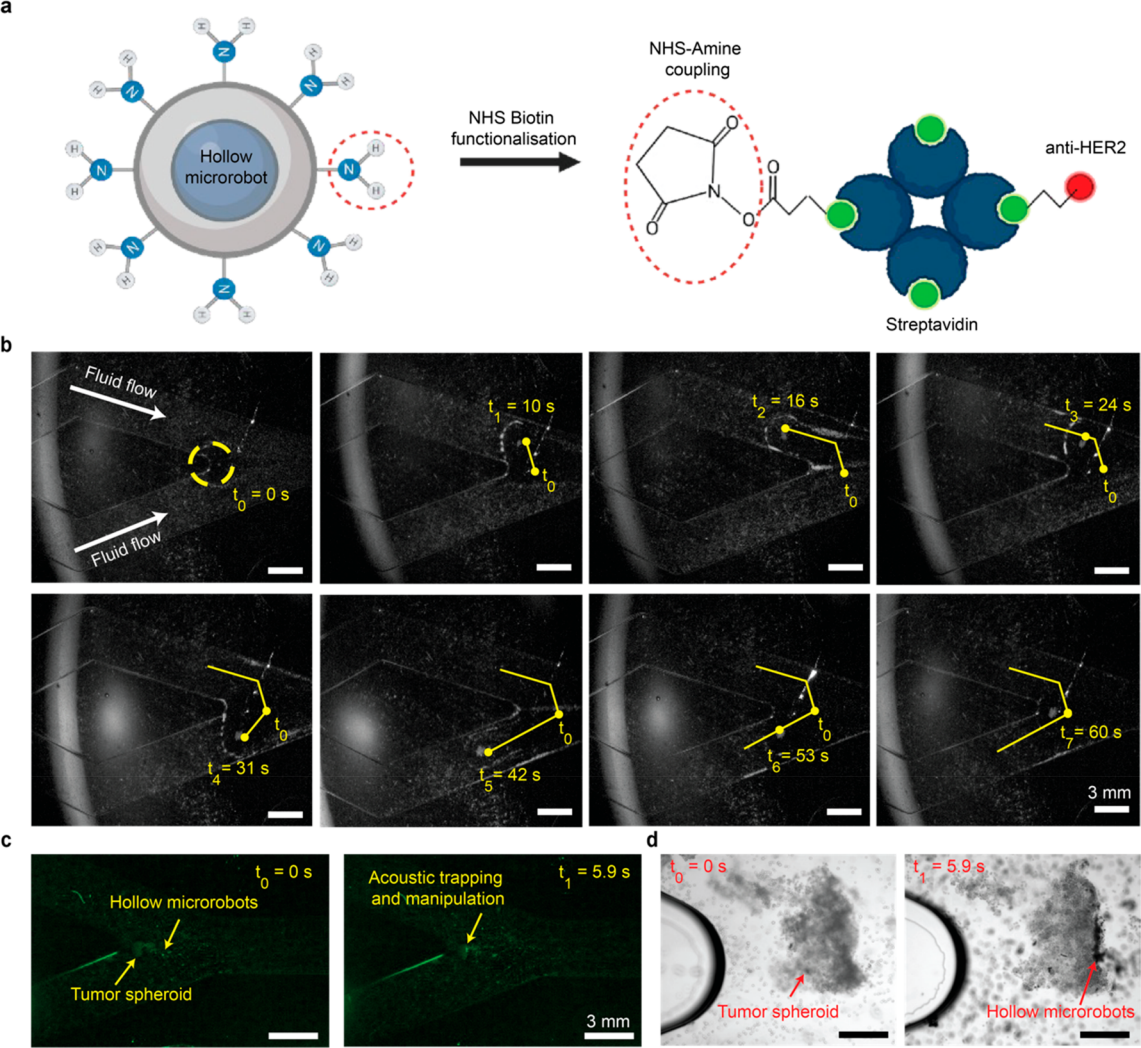
Trapped particle swarm movement inside a bifurcated microfluidic channel and active cell tagging. a) Schematic representation of the functionalization procedure for binding an anti-HER2 antibody to hollow borosilicate particles. First NH2 groups are added to the particles using APTES modification. Subsequently NHS-Biotin and streptavidin are bound to the particles. In the last step, a biotinylated anti-HER2 is bound to the particles using streptavidin–biotin interaction. b) Movement of hollow particles inside a bifurcated microfluidic chip intended for cell culture. Here a flow rate of 0.75 mL/min was applied. The flow direction is depicted by a green arrow. From the starting position t_0_ the particles are moved in the top bifurcation against the flow. Afterward the particles were moved back to the starting position in the direction of the flow. Finally, the particles were moved to the bottom bifurcation and back to the starting point. For acoustic trapping, a focused 2 MHz transducer was used. The current trapping region is marked in each image by a blue dotted circle. c) Microscope images showing the trapping and subsequent movement of hollow particles through a microfluidic channel toward a tumor spheroid (yellow frame). The trapping region is indicated by a purple dotted circle. When near the tumor spheroid the particles attached to the tumor spheroid (pink frame) and escaped the acoustic trap (*t* = 5.93 s). The color table of the microscope images was changed to green for better visibility. d) Light microscope image of SKBR3 cells (immortalized human breast cancer cell line) embedded into a cell culture chip surrounded by a yellow frame. The image surrounded by a pink frame is taken after injection and FUS-enabled movement of anti-HER2 functionalized hollow particles to the cancer spheroid. The particles are bound to the cells by an antibody-receptor interaction.

To demonstrate the potential use of acoustic-trapping-based particle manipulation for active cancer targeting, we conjugated the hollow borosilicate particles with HER-2 antibodies, shown in Figure 5a. HER-2 is overexpressed by many breast cancer cells^22^ and hence a commonly used biomarker for breast cancer targeting. Identifying or separating specific cell types or matter is a commonly used practice in cell and medical research.^44,45^ This is mainly realized in microfluidic chips with a rectangular flow area instead of a circular flow area, as present in the tubings shown before. Consequently, we also present the suitability of the trapping of hollow particles using FUS waves in this scenario.

For future applications, besides the movement of particles against flows, also their controlled movement into bifurcations is crucial. Hence, bifurcated microchannels with a width of 3 mm and a height of 0.4 mm with different flow rates were used to test the precise movement of the particles into both branches of the channel. To allow movement in the *x*-*y*-*z* direction, the channel was mounted to a manual *x*-*y*-*z* stage as used in previous experiments. After the injection of particles into tubing connected to the microchannel, they were transported into the field of view. When the particles were flushed into the microchannel, they were trapped using a 2 MHz FUS transducer. The controlled movement of the particles in and out of the bifurcation was demonstrated under varying flows of 0.25, 0.5, 0.75, and 1 mL/min. Considering the channel dimensions, this corresponds to flow speeds of 34.7–139 mm/s. Successful trapping and movement of particles could be demonstrated until a flow rate of 0.75 mL/min, corresponding to a flow speed of 104.2 mm/s (Figure 5b, Movie S5). As previously described, these flow speeds suffice the requirements for microfluidic applications.^51^ The large difference in flow velocities in cylindrical channels and rectangular channels is attributed to the geometry of the channels. Due to the rectangular shape, more particles can be present at the channel’s walls where they experience significantly less flow than in the middle of the channel. This allows for movement against very high flow speeds.

For the experiments, a concentration of 1 × 10^7^ cells/mL was seeded into a commercially available bifurcated microfluidic chip. The cells were incubated inside the upside-down chip for 2 h. Hence, the cells were attached to the top of the chip, ensuring contact between the breast cancer cells and the buoyant particles after injection into the chip. After injection into the chip, hollow particles were acoustically trapped using a 2 MHz transducer and moved toward a cancer spheroid (Figure 5c, Movie S6). Close to the cancer spheroid, the particles left the acoustic trap and attached to the front side of the cancer spheroid (Figure 5d, Movie S6). This active targeting can have various applications in the field of cell and microbiology as well as biomedical research. Due to the small size of the transducer probe, this technology can easily be incorporated into a benchtop research setup, especially when using a probe with a shorter focal distance.

## Discussion

In this study, we present the use of single-lens FUS waves for the acoustic trapping of hollow microparticles. The proposed hollow microparticles combine the advantages of microbubbles, such as strong ultrasound response, with the rigid shell materials of synthetic microparticles for better stability and control in confined spaces and fluid flows. The acoustic forces allowed local aggregation of hollow microparticles at the focal spot of FUS waves without any coalescence issues that are intrinsic to free microbubbles. The dynamics of the acoustic trapping inside a vessel was characterized, and the trapping performance was evaluated under different flow rates and ultrasound amplitudes. Additionally, we demonstrated a strategy for removing the microparticles by steering the FUS transducer against the flow after a few seconds of injection. Lastly, the potential biomedical application of coated hollow microparticles was demonstrated, specifically in active cell tagging and navigation in bifurcated channels under fluid flow. The results of this study provide a proof of concept for the manipulation of hollow microparticles by using a single-lens FUS transducer.

The presented technology possesses the advantageous attributes of low expense, miniature/compact size, and user-friendly operation, making it amenable to integration with existing ultrasound systems. However, a few issues regarding the focal spot detection of FUS waves in tortuous vessels remain to be investigated. This is a general issue when targeting FUS waves, especially in transcranial applications.^53^ Note that the detection of the FUS focal spot, especially within intricate vascular structures, presents a nontrivial challenge. Additionally, precise control over the applied radiation force and sound pressure intensity is necessary to avoid bursting vessels or heating of tissue. Also, the scattering of bones may be a concern for applications inside the brain or close to the ribs, such as the vascular system around the heart. Nevertheless, this technology has the potential to impact the field of targeted drug delivery by enabling the creation of compact, cost-effective, and portable actuation devices and microparticles. Furthermore, it removes the need for the complex fabrication of advanced FUS transducers.^38,39^ The acoustic manipulation and control of hollow microparticles open a wide array of possibilities for applications in biology, chemistry, physics, and biomedical research. The current experiments indicate that the trapping mechanism is the result of a multifaceted interplay between various forces; however, the precise physics underlying the trapping remains to be fully understood in future work. A subsequent study aims to experimentally isolate and identify the individual mechanisms, such as acoustic streaming, acoustic radiation, particle–particle interactions, and boundary forces, through the utilization of finite element and numerical simulations, which will lead to a more comprehensive understanding of the trapping mechanism. Other areas of research include the integration of image-based feedback control using X-ray, optical, optoacoustic, and acoustic imaging. Additionally, if this method were to be applied to drug carrier systems, then it would be necessary to test its performance in biological fluids, such as blood.^54,55^

There are other materials such as polymeric particles^56,57^ and gas vesicles^58,59^ in the literature with negative acoustic contrast factor. It would be worthwhile to compare the values of the expected negative contrast factor and range of acoustic radiation force between them. For instance, gas vesicles^58^ have an estimated acoustic contrast factor of around −15 with the expected acoustic radiation force in the range of 0.01–10 pN. In our work, the hollow microparticles have a contrast factor of around −0.5 with expected radiation force of up to 4 nN. The size scale of the gas vesicles is in the range of 200–800 nm, whereas the size range of our particles lie between 5 and 20 μm. The larger size indicates a higher acting force on hollow microparticles compared to the gas vesicles, even though the acoustic contrast factor of hollow microparticles is much less than that of the gas vesicles. So far, the main advantage of the current microparticles is their stability compared to that of the gas vesicles. On the other hand, the main disadvantage of these synthetic microparticles is that they cannot be used as genetically encodable actuators inside cells, which limits their use for cell manipulations.

As ultrasound agents, these hollow microparticles not only provide high contrast for deep tissue imaging but also can form stable aggregates at specific locations and be manipulated within the circulatory system while under fluid flow. Furthermore, like other solid microparticles, hollow microparticles have the advantage of high-throughput fabrication and versatile chemical modification capabilities, such as surface coating,^36,60^ drug loading,^61,62^ and cargo attachment.^63^ These drug-loaded microparticles can be trapped with submillimeter accuracy in proximity to diseased tissue and then release the drugs through a series of ultrasound pulses. Additionally, the buoyancy of these hollow microparticles can be adjusted to match the medium in which they are immersed, which eliminates the issue of sedimentation and makes their manipulation with acoustics more manageable. Future research will focus on developing methods to control the long-term attachment of these microparticles to diseased tissue and the optimal methods of drug release using ultrasound.

## Methods

### Scanning Electron Microscope (SEM) Imaging of Hollow Particles

The hollow borosilicate particles were coated with 10 nm of Au in preparation for SEM (Zeiss, Germany) imaging. The Au served as a conductive surface to decrease charging during the imaging procedure. After the acquisition of SEM images of intact hollow particles, the borosilicate shell was cracked. Therefore, a glass slide was positioned parallel to the sample and pressure was applied manually. The cracked particles were imaged subsequently to determine the shell thickness of the hollow borosilicate particles.

### Trapping and Retrieval of Hollow Particles in Bifurcated Channels and Tubings

The setup consisted of an FUS transducer with a central frequency of 2 MHz (SU-101, Sonic Concepts, U.S.A.) or 500 kHz (H-104G, Sonic Concepts, U.S.A.) embedded into a customized glass tank with dimensions of 30 × 30 × 20 cm^3^. The 500 kHz probe is made of stainless steel with 81.79 mm (3.220 in.) diameter × 19.05 mm (0.750 in.) height, and the 2 MHz probe is made of brass with 39.37 mm (1.550 in.) diameter and 12.7 mm (0.500 in.) height. The transducer was fixed to the bottom of the tank using a double-sided adhesive tape and connected to an amplifier (ar, 150A100C, Germany). A function generator (AFG 1022 Tektronix GmbH, Germany) attached to the amplifier generated the input for the amplifier including the input voltage and the frequency. To measure the output signal of the amplifier also an oscilloscope (MDO 4042C Tektronix GmbH, Germany) was connected to it. The first set of experiments was conducted in Tygon tubings with two different diameters. According to the manufacturer (Antylia Scientific, USA), the smaller tubing has an inner diameter of 0.5 and 1.5 mm. The inner diameter of the larger tubing is 4.77 mm and the outer diameter is given by 7.9 mm. The measurements via optical microscope are shown in Figure S11. The whole pig blood was provided by a slaughter house in Ulm, and an anticoagulant, citrate, was added to prevent the clotting. To determine the ability of the focused ultrasound trapping inside a bifurcated channel under flow a cell culture chip was used (μ-slide y-shaped, ibidi, Germany). For precise positioning, the channel was attached to a customized holder connected to an *x*-*y*-*z* stage (Thorlabs Inc., USA). To provide flow inside the channel, tubings were attached to the chip and a syringe pump and sealed using Luer locks (ibidi, Germany). The residue was collected in a reservoir. The particles were injected into the tubing using a 24 G needle and a 1 mL volume syringe.

### Hydrophone Measurements

The setup for these measurements is similar to that described above for the trapping of particles inside a bifurcated channel. To access the pressure applied by the transducer and to characterize the focal point, a needle hydrophone was attached to an *x*-*y*-*z* stage by using rods and a holder (Thorlabs Inc., U.S.A.). The hydrophone is placed over the center of the transducer, while only the tip of the hydrophone is in contact with the water. Connect the hydrophone was connected to an oscilloscope to record the acquired signal. A burst of US was sent with the transducer, and the signal was recorded using the hydrophone. This procedure was repeated for several *x*-*y* positions. The acquired pressure map for different amplitudes and frequency data at each specific point was used to extrapolate for other locations. Finally, the pressure at the focal point of the transducer was also measured for different input voltages. Using the calibration sheet of the hydrophone, the measured voltage was transformed into pressure (Figure S12). The acoustic intensity is calculated by , whereas Δ*p* is the change in pressure, ρ is the density of the medium the acoustic wave traveling through, and *v*_*w*_ is the speed of sound in water. Using this equation, we get a maximum sound intensity of 97.85 mW/mm^2^ for Δ*p* = 0.541 MPa, ρ = 997 kg/m^3^, and *v*_*w*_ = 1500 m/s.

### Numerical Simulations

For numerical simulations first, a 3D fluid dynamics simulation was performed as validated previously.^64^ The corresponding file was set up in COMSOL. As geometries, a tubing with 4.76 mm diameter or 500 μm diameter as well as a sphere with differing diameters (10, 100, and 500 μm) was defined. The position of the sphere inside the tubing was changed during the simulations. Starting from the center of the tube, the sphere was moved closer to the tubing wall. The calculated drag forces for each particle diameter, flow rate, tubing diameter, and relative position of the particle were used as inputs for a numerical simulation of the magnetic gradient required to equalize the drag force and enable successful trapping. The numerical simulations were automatically performed by using a customized and programmed Excel file.

### Ex Vivo Ultrasound Imaging of Hollow Particles

Ultrasonographic examination of the hollow particles was performed on ex vivo liver tissues of mice with liver metastases. The ex vivo mice tissues were shared by the Max Planck Institute for Biology of Aging (Cologne, Germany) after animals were euthanized, and the related tissues were collected for their related research project. At 6 h after euthanasia, the hollow particle suspension was injected into the portal vein of the liver with the help of a 26G needle connected to 1 ml BD Safety-Lok disposable syringe. During the injection, the localization of the needle and the hollow particles was observed with a high-resolution ultrasound imaging system Vevo 3100 (FUJIFILM VisualSonics Inc., Toronto, ON, Canada). The whole procedure of locating the needle into the tissue and the followed hollow particle suspension injection was imaged with an MX Series transducer (MX550D) at the frequency of 40 MHz using the B-Mode and the Vevo 3000 software.

### Functionalization of Hollow Particle Surfaces with HER-2 Antibodies

Commercially available hollow borosilicate particles (Cospheric, U.S.A.) were first functionalized inside an Eppendorf tube using (3-aminopropyl)triethoxysilane 99% (APTES) (Sigma-Aldrich, U.S.A.) diluted to a concentration of 5% inside Ethanol. The Eppendorf was sealed using Parafilm (Sigma-Aldrich) and aluminum foil and placed on a shaker at 2400 rpm overnight. Afterward the particles were placed on a heater at 65 °C for 1h. After washing the particles four times using dimethyl sulfoxide (DMSO) (ThermoFisher, U.S.A.), 5 mg/mL *N*-hydroxysuccinidobiotin (NHS Biotin) (ThermoFisher, U.S.A.) was added to the particles. The particles were sealed again and put into the shaker at 2400 rpm for 3h. Next, particles were washed four times using phosphate-buffered saline solution (PBS) (Sigma-Aldrich, U.S.A.) and 20% (200 μL/ml) of Streptavidin (ThermoFisher, U.S.A.) was added (Figure S13). The particles were placed for 1h in the shaker at 2400 rpm. Subsequently the particles were washed with PBS four times and 50 μL of biotinylated antihuman HER-2 (Sigma-Aldrich, U.S.A.) was added to the particles.

### Breast Cancer Cell Tagging Inside Bifurcated Channels

To demonstrate the ability of HER-2 functionalized borosilicate particles to tag breast cancer cells (SKBR3, Hölzel Diagnostika, Germany) the same setup as described for trapping inside bifurcated channels was used. The chips (μ-slide y-shaped, ibidi, Germany) were first rinsed with ethanol and afterward with sterile PBS under a fume hood. To further decrease the risk of contamination, the chip was placed under UV light for 1 h. Thereby the presence of a PBS meniscus was ensured at the channel opening to avoid evaporation. Next the channels were rinsed with diluted fibronectin (dilution 1:10, 50 μL of fibronectin + 450 μL of PBS) (Sigma-Aldrich, U.S.A.) and placed upside-down inside an incubator at 37 °C for 1 h. The channels were flushed with the cell culture medium to remove unbound fibronectin. Afterward SKBR3 cells were seeded into the chips using a density of 1 × 10^7^ cells/mL. The chips were placed upside down into a Petri dish and placed inside the incubator for 2 h. The orientation of the chip was important to ensure that the particles also were in contact with the cells during passive injection. Since the particles are buoyant, they move to the top of the channel. Consequently, cells were seeded in the top part of the channel. Finally, the channels were flushed with PBS, and microscope images were taken to ensure the attachment of cells.

### Statistical Analysis

All quantitative experimental values are presented as means ± SD of the mean.

## Data Availability

All data are available from the corresponding author upon reasonable request. All relevant codes are available upon request from the corresponding author.

## Supporting Information Available

Size-frequency distribution, cell viability tests for different concentrations of hollow microparticles, sample frame of video with the ROI, trapping dynamics of the hollow particles, diagrams showing the magnetic gradients needed to trap particles, optical measurements of the tube diameters, acoustic pressure amplitude of the FUS transducer, optical densities of streptavidin stock solutions and retention media (PDF)
Movie S1: Acoustic trapping of hollow microparticles against fluid flow using FUS (MP4)
Movie S2: Acoustic trapping of solid vs hollow microparticles (MP4)
Movie S3: Acoustic trapping and retrieval of hollow microparticles (MP4)
Movie S4: Acoustic trapping and manipulation of hollow microparticles under flow (MP4)
Movie S5: Acoustic trapping and manipulation of hollow microparticles in bifurcated microfluidic chip against flow (MP4)
Movie S6: Active tagging of SKBR3 breast cancer cells (MP4)
Movie S7: Ultrasound imaging of hollow particles inside a mouse cadaver (MP4)
