# Supplementary material for: Acoustic Trapping and Manipulation of Hollow Microparticles under Fluid Flow Using a Single-Lens Focused Ultrasound Transducer
Source: ACS Appl Mater Interfaces. 2023 Nov 2;15(45):52224–36. doi: 10.1021/acsami.3c11656 (PMC10658455; doi:10.1021/acsami.3c11656)
Supplement: Supplementary file 6 — am3c11656_si_006.pdf [file am3c11656_si_006.pdf]

Supporting Information

Acoustic trapping and manipulation of hollow microparticles under fluid flow using a single-lens focused ultrasound transducer

Paul Wrede*[1], Amirreza Aghakhani*+[1,2], Ugur Bozuyuk[1], Erdost Yildiz[1], Metin Sitti+[1,3,4]

[1] Physical Intelligence Department, Max Planck Institute for Intelligent Systems, 70569, Stuttgart, Germany
[2] Institute of Biomaterials and Biomolecular Systems, University of Stuttgart, 70569, Stuttgart, Germany
[3] Institute for Biomedical Engineering, ETH Zurich, 8092, Zurich, Switzerland
[4] School of Medicine and School of Engineering, Koç University, Istanbul, 34450, Turkey

Corresponding authors: amirreza.aghakhani@bio.uni-stuttgart.de; sitti@is.mpg.de

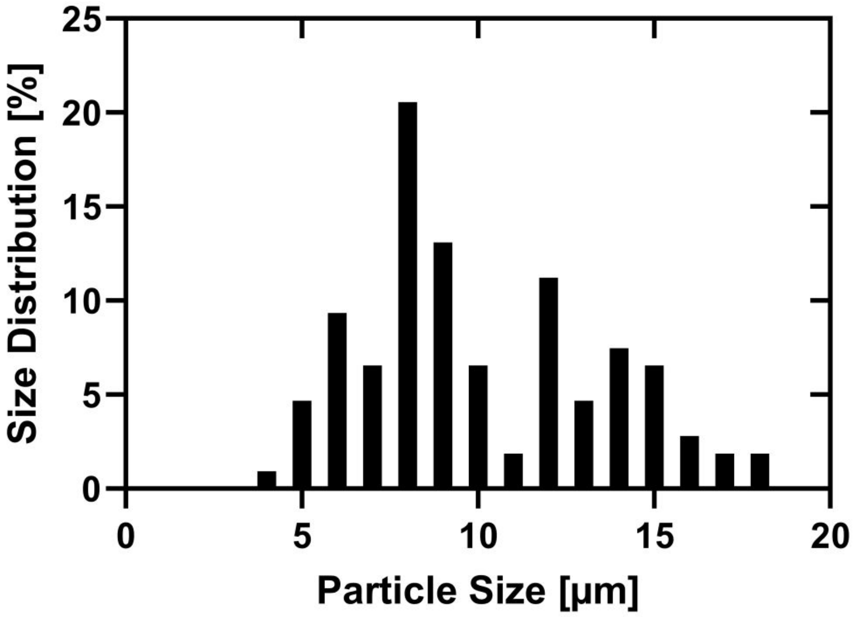

Figure S1. Bar chart showing the size-frequency distribution of hollow particles as used for this study. Here 107 particle diameters were measured from microscope images using ImageJ. Approximately 50% of all particles have a size between 8 and 12 µm.

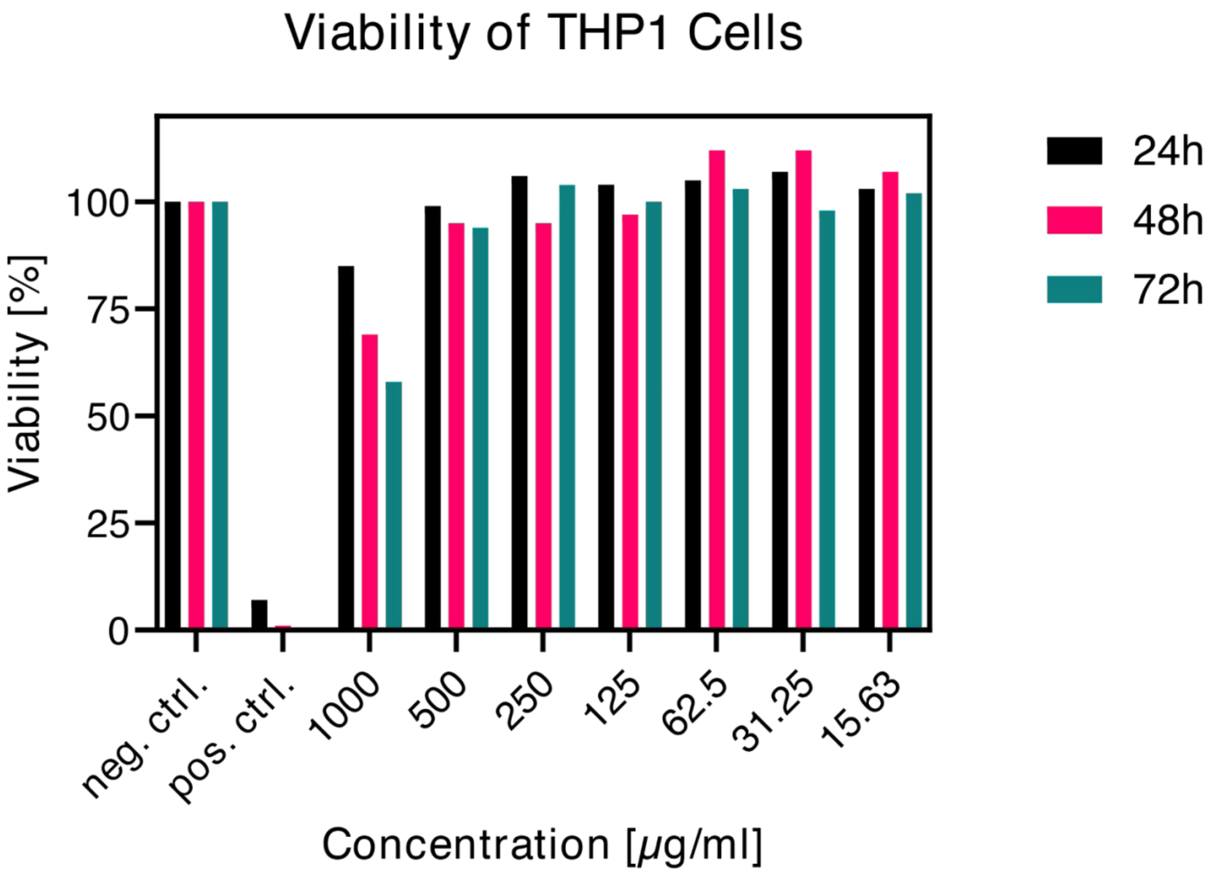

Figure S2. Cell viability tests for different concentrations of hollow microparticles for a duration of 72 hours.

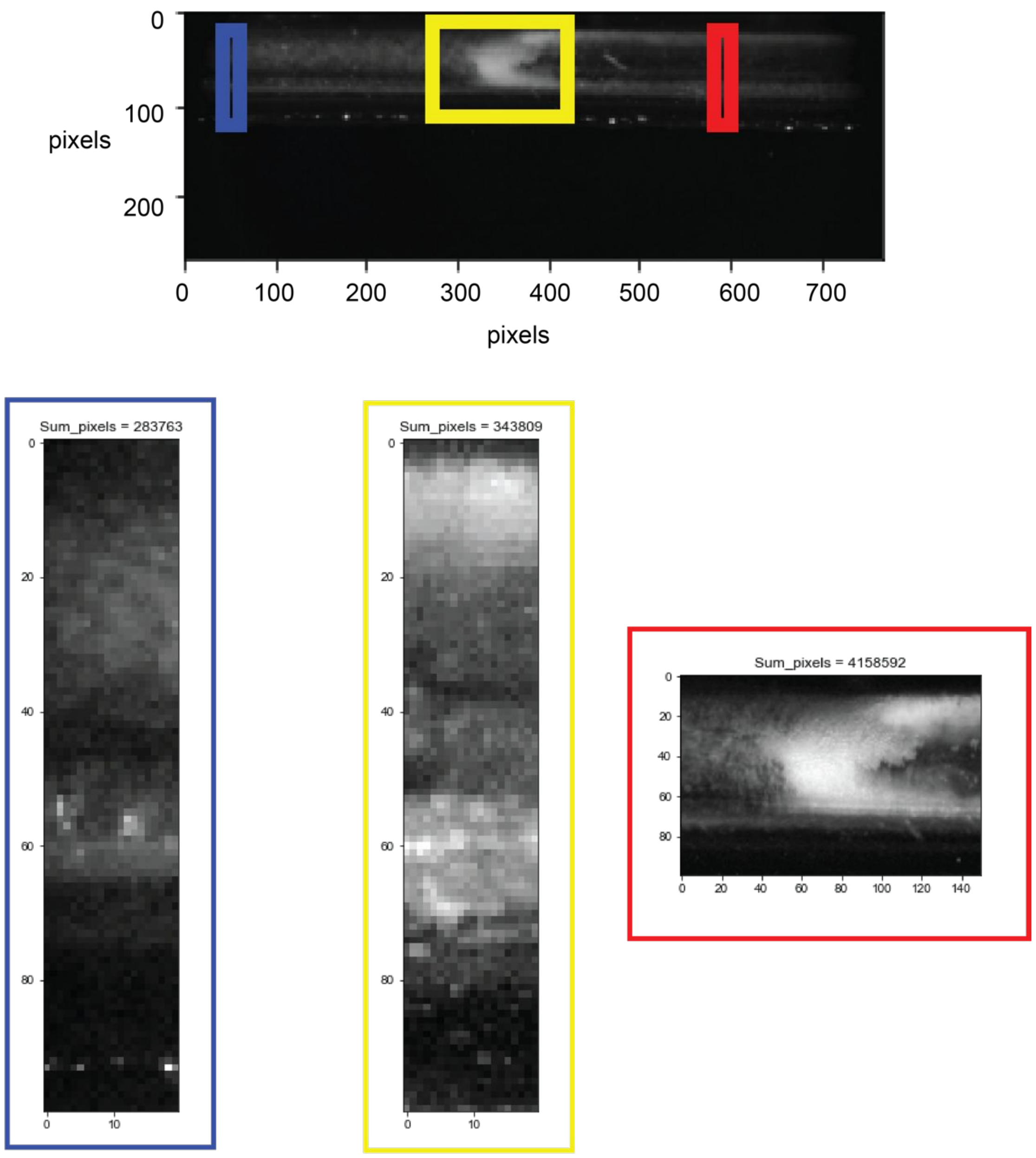

Figure S3. A sample frame of video with the ROI for regions before (blue rectangle), during (yellow rectangle), and after (red rectangle) the acoustic trap. The accumulative pixel intensity in each ROI was used to calculate the normalised concentration of particles over the duration of the experiment.

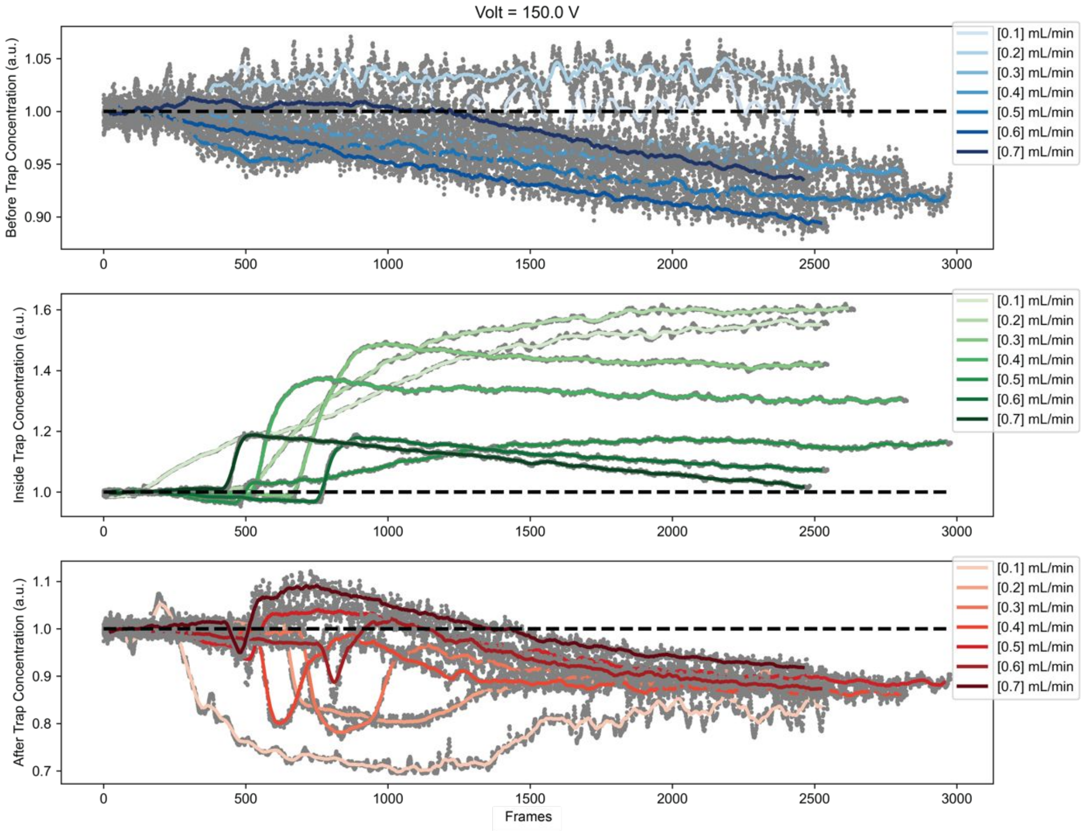

Figure S4. Trapping dynamics of the hollow particles inside a FUS field of 2MHz and 150 V voltage input. The plots show the normalized particle concentration before, inside, and after the trapping region over time for flow rates reaching from 0.1 to 0.7 ml/min. Each test was recorded at 400 frames per second.

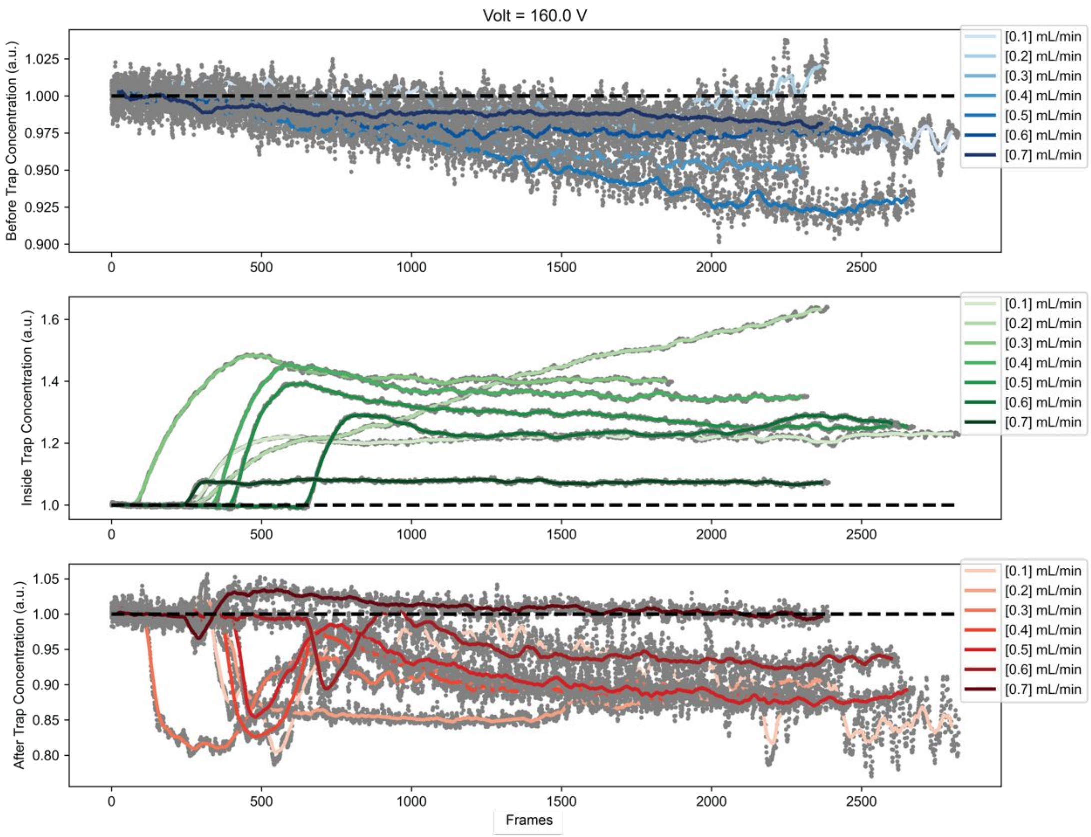

Figure S5. Trapping dynamics of the hollow particles inside a FUS field of 2MHz and 160 V voltage input. The plots show the normalized particle concentration before, inside, and after the trapping region over time for flow rates reaching from 0.1 to 0.7 ml/min. Each test was recorded at 400 frames per second.

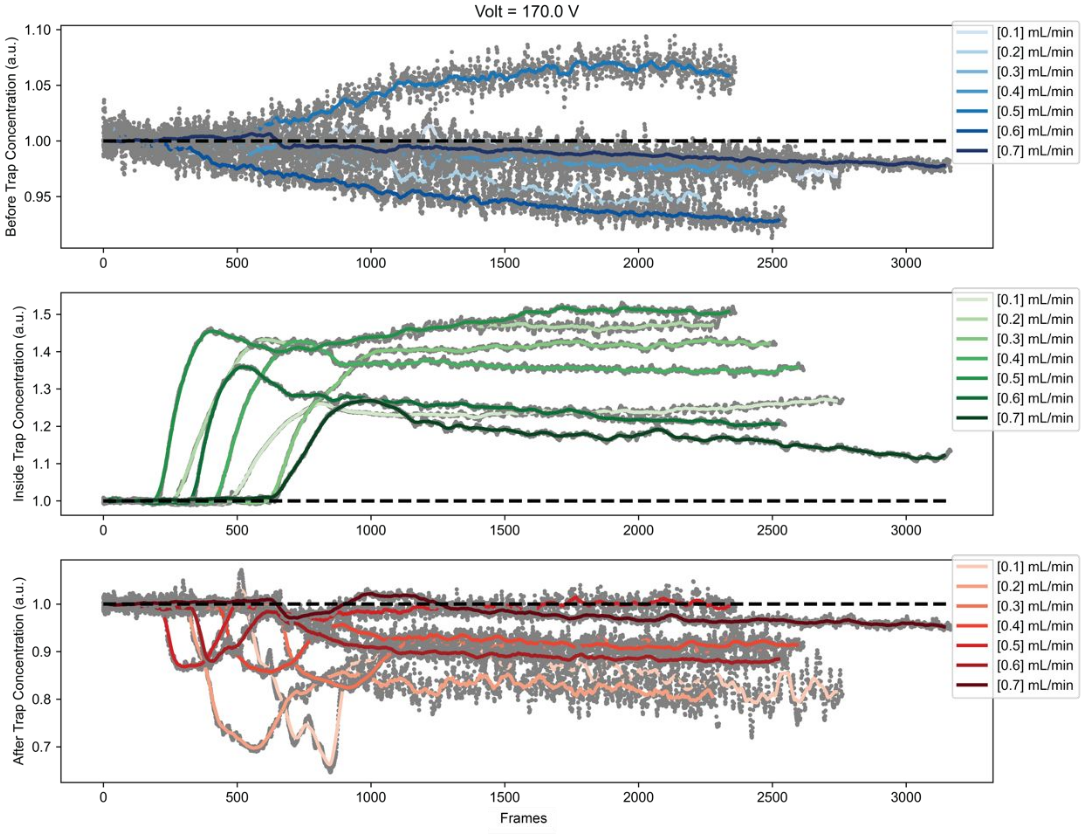

Figure S6. Trapping dynamics of the hollow particles inside a FUS field of 2MHz and 170 V voltage input. The plots show the normalized particle concentration before, inside, and after the trapping region over time for flow rates reaching from 0.1 to 0.7 ml/min. Each test was recorded at 400 frames per second.

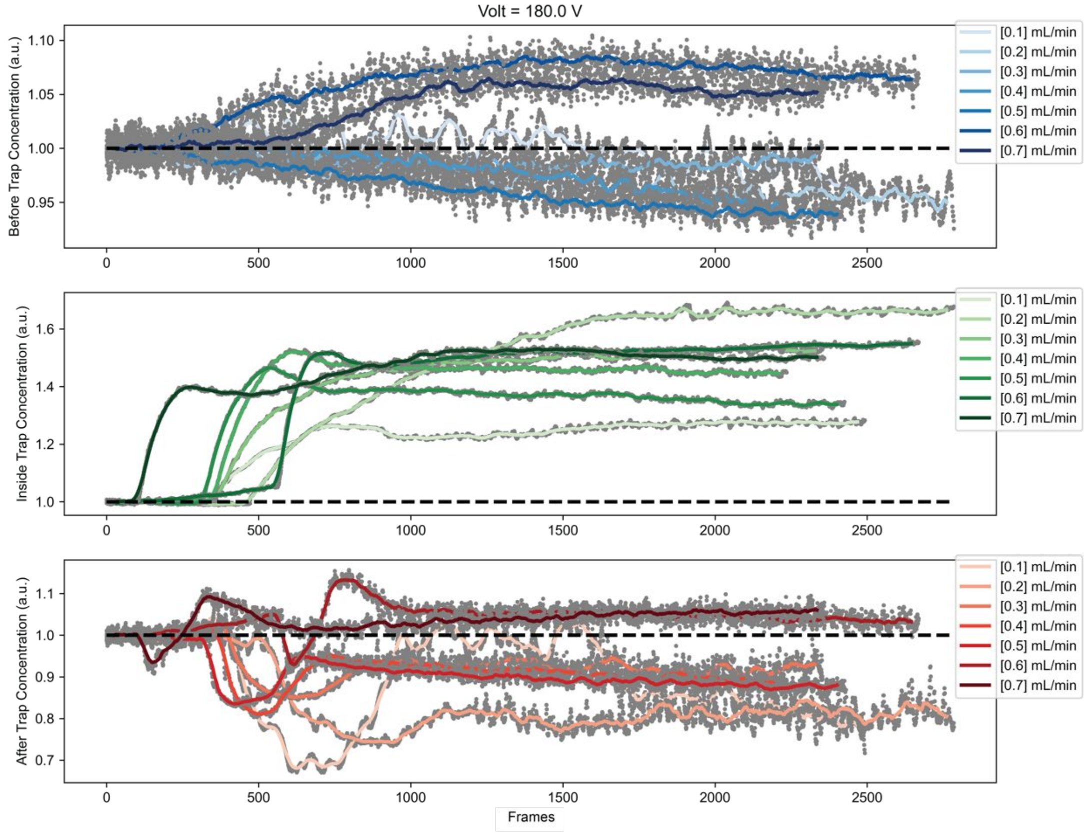

Figure S7. Trapping dynamics of the hollow particles inside a FUS field of 2MHz and 180 V voltage input. The plots show the normalized particle concentration before, inside, and after the trapping region over time for flow rates reaching from 0.1 to 0.7 ml/min. Each test was recorded at 400 frames per second.

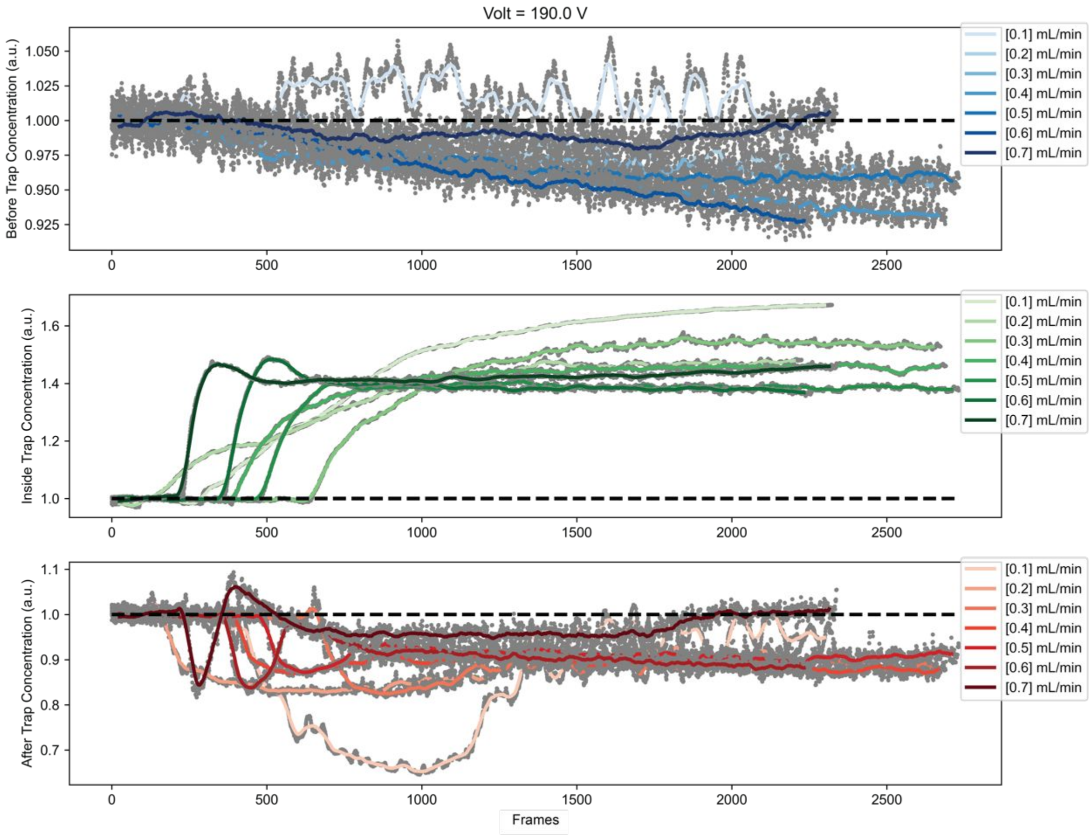

Figure S8. Trapping dynamics of the hollow particles inside a FUS field of 2MHz and 190 V voltage input. The plots show the normalized particle concentration before, inside, and after the trapping region over time for flow rates reaching from 0.1 to 0.7 ml/min. Each test was recorded at 400 frames per second.

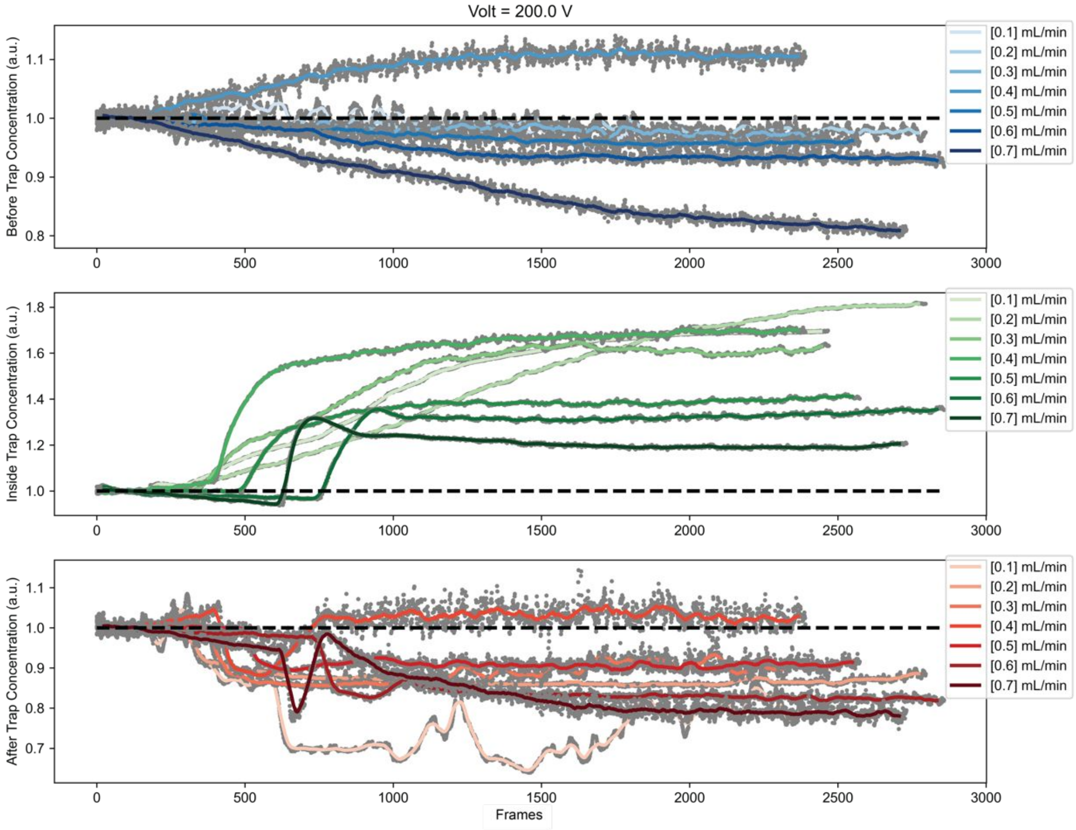

Figure S9. Trapping dynamics of the hollow particles inside a FUS field of 2MHz and 200 V voltage input. The plots show the normalized particle concentration before, inside, and after the trapping region over time for flow rates reaching from 0.1 to 0.7 ml/min. Each test was recorded at 400 frames per second.

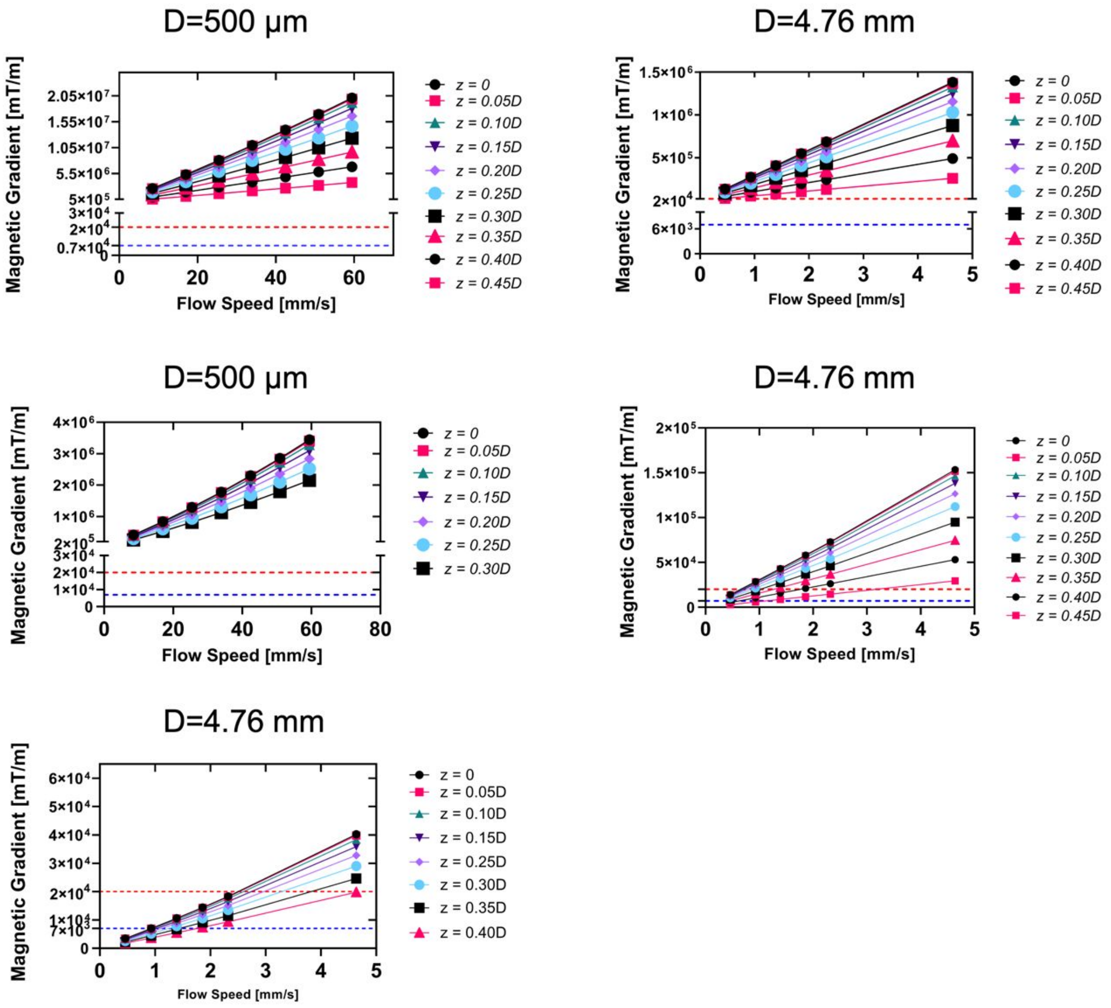

Figure S10. The diagrams show the magnetic gradients needed to trap particles under several conditions, inside different vessel diameters (D).

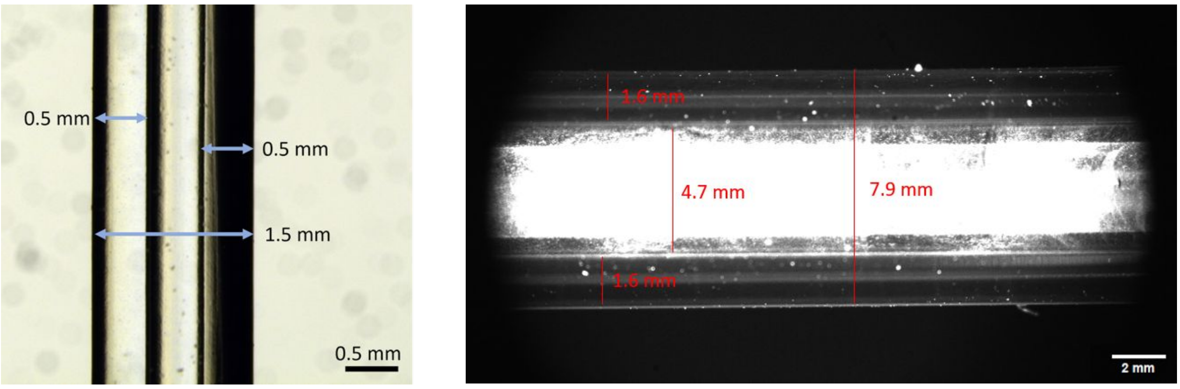

Figure S11. The optical measurements of the tube diameters. The 0.5-mm-diameter tube shown on the left and 4.7-mm-diameter tube shown on the right panel.

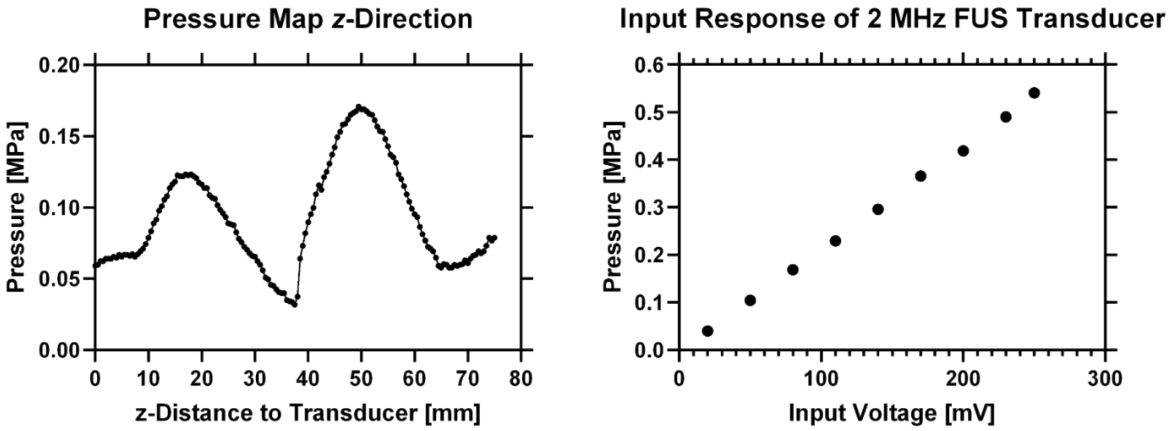

Figure S12. The acoustic pressure amplitude of the FUS transducer with vertical distance at the focal point (left) and versus the input voltage (right)

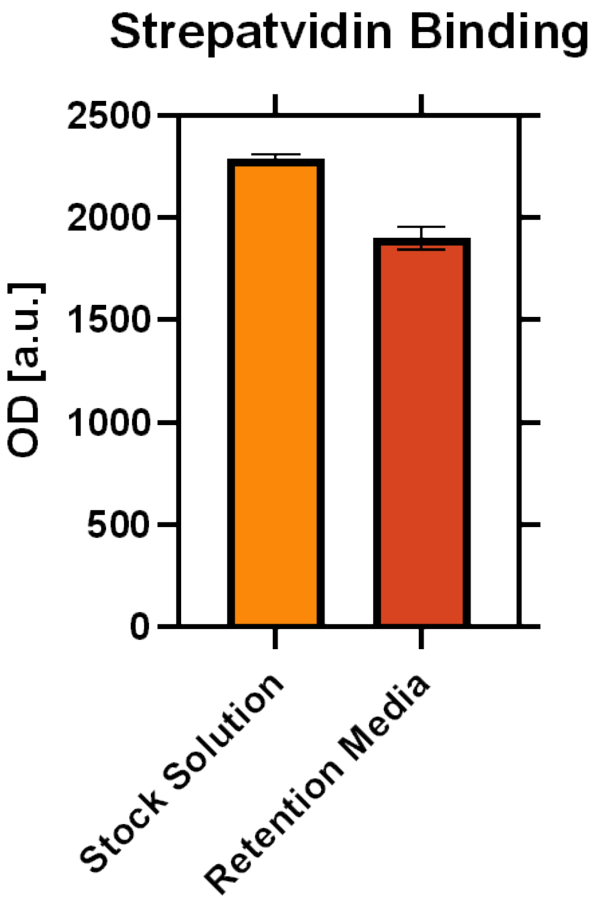

Figure S13. The optical density (OD) of 100 μL of the streptavidin stock solution and 100 μL of the retention media following particle conjugation with Streptavidin, Biotin, and Anti-HER2
